# Effects of Probiotics and Synbiotics on Obesity, Insulin Resistance Syndrome, Type 2 Diabetes and Non-Alcoholic Fatty Liver Disease: A Review of Human Clinical Trials

**DOI:** 10.3390/ijms17060928

**Published:** 2016-06-13

**Authors:** Maria Jose Sáez-Lara, Candido Robles-Sanchez, Francisco Javier Ruiz-Ojeda, Julio Plaza-Diaz, Angel Gil

**Affiliations:** 1 Department of Biochemistry & Molecular Biology I, School of Sciences, University of Granada, Granada 18071, Spain; mjsaez@ugr.es; 2 Institute of Nutrition and Food Technology “José Mataix”, Biomedical Research Center, University of Granada, Armilla 18100, Spain; croblesan@hotmail.com (C.R.-S.); fruizojeda@ugr.es (F.J.R.-O.); jrplaza@ugr.es (J.P.-D.); 3 Department of Biochemistry and Molecular Biology II, School of Pharmacy, University of Granada, Granada 18071, Spain; 4 Instituto de Investigación Biosanitaria ibs, GRANADA, Complejo Hospitalario Universitario de Granada/Universidad de Granada, Granada 18014, Spain; 5 CIBEROBN (Physiopathology of Obesity and Nutrition CB12/03/30038), Instituto de Salud Carlos III (ISCIII), Madrid 28029, Spain

**Keywords:** probiotics, randomized clinical trial, obesity, insulin resistance, metabolic syndrome X, type 2 diabetes, non-alcoholic fatty liver disease, synbiotics

## Abstract

The use of probiotics and synbiotics in the prevention and treatment of different disorders has dramatically increased over the last decade. Both probiotics and synbiotics are well known ingredients of functional foods and nutraceuticals and may provide beneficial health effects because they can influence the intestinal microbial ecology and immunity. The present study reviews the effects of probiotics and synbiotics on obesity, insulin resistance syndrome (IRS), type 2 diabetes (T2D) and non-alcoholic fatty liver disease (NAFLD) in human randomized clinical trials. Select probiotics and synbiotics provided beneficial effects in patients with obesity, mainly affecting the body mass index and fat mass. Some probiotics had beneficial effects on IRS, decreasing the cell adhesion molecule-1 levels, and the synbiotics decreased the insulin resistance and plasma lipid levels. Moreover, select probiotics improved the carbohydrate metabolism, fasting blood glucose, insulin sensitivity and antioxidant status and also reduced metabolic stress in subjects with T2D. Some probiotics and synbiotics improved the liver and metabolic parameters in patients with NAFLD. The oral intake of probiotics and synbiotics as co-adjuvants for the prevention and treatment of obesity, IRS, T2D and NAFLD is partially supported by the data shown in the present review. However, further studies are required to understand the precise mechanism of how probiotics and synbiotics affect these metabolic disorders.

## 1. Introduction

The microbiota (the full collection of microbes that naturally exist within a particular biological niche) has a profound influence on human physiology, affecting metabolism and the immune system and protecting against pathogens while modulating gastrointestinal (GI) development [1,2,3]. Perturbations in the composition of the microbiota may be especially important during early life, when the immune system is still developing [4].

Obesity is one of the most important public health problems worldwide, affecting both developed and emerging countries. It is characterized by an abnormal excess of white adipose tissue, which is a major risk factor for the development of diabetes, cardiovascular disease and cancer [5,6]. Among the studied potential determinants of obesity, the intestinal microbiota has been proposed to have an impact on the energy balance in humans [7,8,9,10,11]. Specific bacterial populations, such as *Prevotellaceae*, *Blautia coccoides*, *Eubacteria rectale* group, *Lactobacillus*, and *Bifidobacterium*, have been reported to be related to obesity. Consequently, it is believed that modulation of the intestinal microbiota toward a healthier “non-obese” profile might present a promising tool for prevention [12].

Metabolic syndrome is referred to as syndrome X or insulin resistance syndrome (IRS). Currently, the number of individuals with IRS is increasing worldwide and is primarily composed of those with abdominal obesity, glucose intolerance, dyslipidemia and high blood pressure. IRS increases cardiovascular morbidity and mortality as well as overall mortality [13]. Low insulin sensitivity, particularity in the visceral adipose tissue, leads to increased free fatty acid flux and inhibition of insulin action in insulin-sensitive tissues. Moreover, IRS is also associated with the development of diabetes mellitus [13,14].

There are about 382 million people living with diabetes in the world, and the number is expected to rise to 592 million by 2035 according to the International Diabetes Federation. The pathogenetic mechanism of type 2 diabetes (T2D) has not yet been completely elucidated. Recent evidence suggests that the intestinal microbiota composition is associated with the development of T2D [15]. A clear relationship has been demonstrated between T2D and compositional changes in the gut microbiota, with a lower relative abundance of *Firmicutes* and a higher proportion of *Bacteroidetes* and *Proteobacteria* in T2D patients [16,17].

Non-alcoholic fatty liver disease (NAFLD) is the most common type of liver disease worldwide that affects both adults and children. NAFLD encompasses a broad spectrum of diseases from simple steatosis at early stages to non-alcoholic steatohepatitis (NASH) and fibrosis, and it eventually may lead to cirrhosis. NAFLD may be caused mainly by obesity, but it can also be caused by T2D, glucocorticoids, and small bowel bacterial overgrowth (SIBO) caused by inflammatory bowel disease. Considering the intimate relationship between the intestine and the liver (called the gut-liver axis), it is becoming widely accepted that the composition of the gut microbiota is an environmental factor that affects host metabolism and contributes to the associated pathological conditions, such as obesity [18].

Probiotics are live microorganisms that confer a health benefit on the host when administered in adequate amounts [19], although dead bacteria and their components can also show probiotic properties. *Bifidobacterium* and *Lactobacillus* strains are the most widely used bacteria exhibiting probiotic properties and are included in many functional foods and dietary supplements [20]. Major mechanisms underlying the antagonistic effects of probiotics include improvement of the gut barrier function, increased competitive adherence to the mucosa and epithelium, gut microbiota modification, and regulation of the gut associated lymphoid immune system. In this sense, probiotics communicate with the host through intestinal cell pattern recognition receptors, such as toll-like receptors and nucleotide-binding oligomerization domain-containing protein-like receptors, which modulate important key signaling pathways, such as nuclear factor-κB and mitogen-activated protein kinase, to enhance or suppress activation and influence downstream pathways [21,22,23,24,25].

A prebiotic is a nonviable food component that confers a health benefit on the host associated with modulation of the microbiota, they may be a fiber, but a fiber is not necessarily a prebiotic. Using prebiotics and probiotics in combination is often described as synbiotic, only if the net health benefit is synergistic [26].

Probiotics and synbiotics are consumed in many and diverse forms, such as yogurt and other fermented milks, cheese and several fermented foods, and also as prevention and treatment for different GI tract dysfunctions and other diseases like allergy [27]. However, the actual effects of probiotics to affect intestinal ecology is still under debate because there are numerous confounding elements, such as dissimilarities in microbial strains, concentrations of viable cells and product formulations [3,28,29].

Recently, Yoo and Kim (2016) [30] have indicated that probiotics and prebiotics affect T2D and cardiovascular diseases by changing gut microbiota, regulating insulin signaling, and lowering cholesterol.

The present review was conducted to investigate the effectiveness of probiotics and synbiotics in the prevention and treatment of obesity, IRS, T2D and NAFLD in human studies designed as clinical trials.

## 2. Results

### 2.1. Effects of Probiotics and Synbiotics on Obesity

#### 2.1.1. Probiotics

*Lactobacillus salivarius* Ls-33 was tested in obese adolescents to investigate its impact on fecal microbiota and anthropometric measures, biomarkers related to inflammation, carbohydrate and lipid metabolism. The ratios of *Bacteroides*, *Prevotellae*, and *Porphyromonas* group bacteria to *Firmicutes*-belonging bacteria, including *Clostridium cluster* XIV, *Blautia coccoides*, *Eubacterium rectale* group and *Roseburia intestinalis*, were significantly increased after the administration of *L. salivarius* Ls-33. However, the overall cell numbers of fecal bacteria, including the groups above as well as *Clostridium cluster* I and cluster IV, *Faecalibacterium prausnitzii*, *Enterobacteriaceae*, *Enterococcus*, *Lactobacillus* group and *Bifidobacterium* spp., were not significantly altered by intervention. Similarly, the short chain fatty acids (SCFA) remained unaffected [31]. Moreover, Gobel *et al.*, (2012) [32] carried out an intervention to study the *Lactobacillus salivarius* Ls-33 effects on some inflammation biomarkers and several parameters of metabolic syndrome in adolescents with obesity. However, they did not find changes in those parameters.

The effect of *L. gasseri* SBT2055 was examined in two studies using a cohort of Japanese adults with large visceral fat areas (VFA). The participants were randomly assigned to three groups receiving increasing colony-forming units (CFUs) of *L. gasseri* SBT2055 for 12 weeks. The results showed a reduction in body mass index (BMI), waist, abdominal VFA and hip circumferences [33,34]. Additionally, a hypocaloric diet supplemented with a probiotic-enriched cheese containing *Lactobacillus plantarum* reduced the BMI, the putrescine content and the intestinal lactobacilli in Russian adults with obesity and hypertension. Similarly, there was a reduction in the blood pressure (BP), namely a lower diastolic BP and a tendency toward lower systolic BP at the end of the intervention in the obese hypertensive patients that received the hypocaloric diet supplemented with the probiotic cheese [35].

The administration of *L. acidophilus* La5, *B. lactis* Bb12, and *L. casei* DN001 was evaluated in individuals with high BMI who were randomly assigned to three groups depending on particular intervention diets: one group was established with a regular yogurt with low calorie diet (RLCD), the second one received a probiotic yogurt with low calorie diet (PLCD) and, the third one received probiotic yogurt without low calorie diet (PWLCD) for about two months. A reduction in BMI, fat percentage, and leptin level was observed that was more obvious in groups who received the weight-loss diet including probiotic yogurt. Additionally, a reduction in the serum levels of CRP was more evident in the PWLCD group than in the PLCD and RLCD groups after the 8-week intervention. The expression of the *FOXP3*, *T-bet*, *GATA3*, *TNF-α*, *IFN-γ*, *TGF-β*, and *ROR-γt* genes in peripheral blood mononuclear cell (PBMCs) were assessed before and after intervention. In the three groups, *ROR-γt* expression was reduced and *FOXP3* was increased. The expression of the *TNF-α*, *TGF-β*, and *GATA3* genes did not change. Interestingly, *T-bet* gene expression was down-regulated in the PLCD and PWLCD groups. However, the *IFN-γ* expression was down-regulated in all groups. The authors suggested that weight loss diet and probiotics yogurt had effects on gene expression in PBMCs among overweight and obese individuals [36,37,38].

An 8-week, randomized, double-blind, placebo- and compliance-controlled parallel study in overweight and obese subjects was conducted to evaluated the effects of one strain of *E. faecium* and two strains of *S. thermophilus* [39]. The patients were randomly divided into five groups: (1) a yogurt fermented with two strains of *S. thermophilus* and two strains of *L. acidophilus*; (2) a placebo yogurt fermented with delta-acid-lactone; (3) a yogurt fermented with two strains of *S. thermophilus* and one strain of *L. rhamnosus*; (4) a yogurt fermented with one strain of *E. faecium* and two strains of *S. thermophiles*; and finally (5) two placebo pills daily [39]. After adjustment for small changes in body weight, low-density lipoprotein cholesterol (LDL-C) decreased and fibrinogen increased significantly after 8 weeks in the group that received the yogurt fermented with one strain of *E. faecium* and two strains of *S. thermophilus* compared to the group consuming chemically fermented yogurt and the placebo pill group. Additionally, after 8 weeks, the systolic BP was significantly more reduced in group 1 and the group that received the yogurt fermented with one strain of *E. faecium* and two strains of *S. thermophilus* than in group 3 [39].

The administration of capsules with bifidobacteria, lactobacilli, and *S. thermophilus* was conducted in overweight subjects. The probiotic mixture had a significant improvement in their lipid profiles, reducing total cholesterol (TC), triacylglycerols (TAG), and LDL-C levels and increasing high-density lipoprotein cholesterol (HDL-C) levels. The probiotic mixture improved insulin sensitivity and decreased C-reactive protein (CRP) [40].

On the other hand, a total of 58 obese, postmenopausal women were randomized into a single-blinded, parallel-group intervention of 6-week duration, with a daily ingestion of *L. paracasei* F19, flaxseed mucilage or placebo. The intake of *L. paracasei* F19 did not modulate any metabolic markers (homeostasis model assessment of insulin resistance (HOMA-IR), Matsuda index, CRP, and lipid profile) compared with the placebo [41]. Moreover, the intake of *L. acidophilus* La5 and *B. animalis* subsp. *lactis* Bb12 not affected the HOMA-IR, BP, heart rate or serum lipid concentrations in overweight adults [42,43].

#### 2.1.2. Synbiotics

The impact of *L. rhamnosus* CGMCC1.3724 with oligo-fructose and inulin supplementation was investigated on weight loss and maintenance in obese men and women during 24 weeks [12].

The mean weight loss in women in the *L. rhamnosus* group was significantly higher than in women in the placebo group after the first 12 weeks, whereas it was similar in men in the two groups. The *L. rhamnosus*-induced weight loss in women was associated not only with significant reductions in fat mass and circulating leptin concentrations but also with the relative abundance of bacteria of the *Lachnospiraceae* family in the feces; this family belongs to the *Firmicutes* phylum, a taxonomic group that has previously been reported to be positively associated with obesity [12].

In obese children, two studies determined the effect of synbiotic supplementation on cardiometabolic risk factors, anthropometric measurements, serum lipid profile, and oxidative stress levels. The intake of synbiotics resulted in a significant reduction in the BMI *z*-score and waist circumference, as well as in some cardiometabolic risk factors, such as TC, LDL-C and TAG [36,37], and also changes in anthropometric measurements (% reduction comparing to baseline) were significantly higher in the children receiving synbiotics. After synbiotic supplementation, total oxidative stress serum levels significantly decreased [44,45].

In summary, the supplementation of selected probiotics appears to have beneficial effects on BMI, waist circumference, VFA and hip circumference in overweight or obese people. Moreover, it has been reported that some probiotic strains modulate the gene expression of specific transcription factors such as *ROR-γt* (down-regulated) and *FOXP3* (up-regulated) in PBMCs. This was associated with beneficial effects on the immune system among overweight and obese individuals. However, no effects were observed at the level of inflammatory biomarkers in the administration of *L. paracasei* F19, and *L. acidophilus* La5 and *B. animalis* subsp. *lactis* Bb12. On the other hand, some synbiotics can reduce BMI in women, decrease fat mass and serum leptin levels and increase the levels of the *Lachnospiraceae* family in the feces. Finally, synbiotic treatment would help to decrease the BMI *z*-score and waist circumference in children, as well as TC, LDL-C and TAG serum levels. Table 1 summarizes the studies of probiotics and synbiotics in obesity.

### 2.2. Effects of Probiotics and Synbiotics on Insulin Resistance Syndrome

#### 2.2.1. Probiotics

The effects of *Lactobacillus casei* Shirota were evaluated in patients with IRS for studying the gut permeability, the presence of endotoxin and neutrophil function, the insulin sensitivity index, quantitative insulin sensitivity check index, insulin sensitivity by oral glucose tolerance test, HOMA-IR and β-cell function. The gut permeability increases, but the endotoxin and neutrophil function remain unaffected [46]. The only parameter that improved after probiotic administration was insulin sensitivity index [47]. IRS in postmenopausal women is an important risk factor for cardiovascular morbidity, especially stroke and coronary heart disease and mortality. The effectiveness of *L. plantarum* or placebo was evaluated in postmenopausal women over the course of 90 days. The TC, interleukin (IL)-6 and γ-glutamyltranspeptidase (γ-GTP) levels were significantly decreased in both groups at the end of the study, whereas the LDL-C level was significantly lower in the placebo group. Glucose and homocysteine levels were significantly reduced in the *L. plantarum* group compared with the placebo group [48].

#### 2.2.2. Synbiotics

Patients with IRS were supplemented with either synbiotic capsules containing seven strains plus fructo-oligosaccharide or placebo capsules to evaluate the insulin resistance and lipid profile. Levels of fasting blood sugar and insulin resistance were improved significantly in the synbiotic group [49].

In summary (Table 2), some probiotic strains have beneficial effects such as decreasing the cell adhesion molecule-1 levels. Moreover, in postmenopausal women, *L. plantarum* decreased TC, IL-6 γ-GTP, glucose and homocysteine levels after 90 days. Finally, synbiotic mixture improved the insulin resistance and HDL-C and reduced the TAG and TC levels in subjects with IRS.

### 2.3. Effects of Probiotics and Synbiotics in Type 2 Diabetes

#### 2.3.1. Probiotics

Hariri *et al.* (2015) investigated the effect of consuming probiotic soy milk containing *L. plantarum* A7 or soy milk alone. Each day, the subjects received probiotic soy milk or regular soy milk to supplement their usual diet. Probiotic soy milk significantly decreased the levels of promoter methylation in the proximal and distal *MLH1* promoter region compared with the baseline values, while the plasma concentration of 8-hydroxy-20-deoxyguanosine decreased significantly compared with the subjects receiving soy milk. Additionally, a significant increase in superoxide dismutase activity was observed in the probiotic soy milk group compared with the baseline value. However, there were no significant changes from baseline in the promoter methylation of *MSH2* within either group. Therefore, *L. plantarum* A7-inoculated soy milk can have antioxidative properties and decrease the risk of mismatched base pairs in DNA among patients with T2D [50].

*L. acidophilus* La-5 and *B. animalis* subsp. *lactis* BB-12 administration was evaluated on T2D patients. There was a significant difference between groups concerning mean changes in the HbA1c, TC and LDL-C levels [51]. In addition, an increase in HDL-C levels and a decrease in the LDL-C/HDL-C ratio in the intervention group [52].

Previous studies using the same strains [53,54] were carried out in T2D patients. The authors reported significantly decreased fasting blood glucose, TC, LDL-C and hemoglobin A1c levels as well as increased erythrocyte superoxide dismutase and glutathione peroxidase activity and total antioxidant status compared with the control group. They concluded that probiotic yogurt is a promising tool for diabetes management, and it may be a functional food that can exert antidiabetic and antioxidant properties [53,54].

In a double-blind, randomized study, males with T2D, impaired or normal glucose tolerance were enrolled and placed on a 4-week treatment course with either *L. acidophilus* NCFM or a placebo to investigate the effects of oral supplementation with the probiotic on insulin sensitivity and the inflammatory response [55]. After the treatment, the probiotic strain was detected in 75% of the fecal samples. Insulin sensitivity was preserved only among volunteers in the *L. acidophilus* NCFM group. Finally, baseline inflammatory markers and the systemic inflammatory response were unaffected by the *L. acidophilus* NCFM supplementation [55].

#### 2.3.2. Synbiotics

The synbiotic supplementation was used to determine the effects on metabolic profiles, CRP and oxidative stress in T2D patients. The multispecies probiotic supplement consisted of 7 viable and freeze-dried strains and fructo-oligosaccharides. A significant increase in HOMA-IR in both groups was detected. However, the increase in the placebo group was significantly higher than that in the probiotic group. The mean changes in serum CRP were significantly lower in the experimental group. Additionally, probiotic supplementation led to a significant increase in the plasma total glutathione levels compared to the placebo [56].

A clinical trials were performed in T2D patients to examined the effects of synbiotic bread contained *L. sporogenes* and inulin. Consumption of the synbiotic bread resulted in a significant reduction in the serum insulin levels, HOMA-IR, and homeostatic model assessment-β-cell function, serum lipid profile (TAG, TC/HDL-C) and a significant increase in the serum HDL-C levels compared to the control bread [57,58].

Finally, a synbiotic shake containing *L. acidophilus*, *B. bifidum* and fructo-oligosaccharides was investigated on glycemic and cholesterol levels in elderly people with T2D. The TC, TAG, HDL-C and blood sugar levels were evaluated. The synbiotic group did not experience any reduction in total cholesterol and TAG levels, but had a significant increase in HDL-C and a significant reduction in fasting glycemia [59].

In summary (Table 3), some of the effects of probiotics in subjects with T2D included a lower fasting blood glucose level, improved insulin sensitivity and an increased antioxidant status. Some synbiotics increased the total glutathione levels, HDL-C and reduced the fasting glycemia levels and CRP. Moreover, an improved serum lipid profile was observed in patients with T2D after the consumption of synbiotics.

### 2.4. Effects of Probiotics and Synbiotics in Non-Alcoholic Fatty Liver Disease

#### 2.4.1. Probiotics

The effects of *L.* *bulgaris* and *S. thermophilus* were measured on different parameters of liver function and cardiovascular risk factors. This treatment revealed a decrease in alanine amino transferase (ALT), aspartate amino transferase (ASP) and γ-GTP levels as indicators of improving liver function [60].

On the other hand, in obese children with NAFLD treated with *L. rhamnosus* strain GG, presented a significant decrease in the titer of anti-peptidoglycan-polysaccharide antibodies, which are suitable as an indirect indicator of SIBO. Moreover, in this randomized clinical trial, a restoration of liver function was also observed in the probiotic group through a decrease in ALT [61].

A double-blind, randomized, controlled clinical trial was conducted using a probiotic yogurt with *L. acidophilus* La5 and *B. lactis* Bb12 for 8 weeks on patients with NAFLD. *L. acidophilus* La5 and *B. lactis* Bb12 consumption resulted in a reduction in the serum levels of ALT, ASP, TC, and LDL-C compared with the control group [62].

In another randomized study, Alisi *et al.* (2014) found a significant improvement in fatty liver severity (evaluated by ultrasound) and a significant decrease in the BMI of children with NAFLD who were treated with bifidobacteria, lactobacilli, and *S. thermophilus* strains for 4 months. These data suggest that those strains might reduce liver fat and therefore prevent the progression of NAFLD [63].

Furthermore, Alisi *et al.* (2014) [63] also evaluated glucagon-like peptide 1 (GLP-1), which is an incretin secreted by cells from the small intestine and proximal colon. GLP-1 is dependent on the presence of nutrients in the lumen of the small intestine. Its physiological effects include the activation of catabolism via an increase in insulin secretion and the suppression of glucagon secretion [63,64]. In this sense, Alisi *et al.* (2014) [63] showed that the circulating levels of the total and active forms of GLP-1 were significantly elevated in the patients after 4 months of treatment with synbiotics. Although there is not yet an adequate amount of data, in humans it has been observed that the use of probiotics improves the efficacy of lifestyle modifications in obese individuals with NAFLD, may improve conventional liver function tests, and may decrease markers of lipid peroxidation [60,61,63,65,66] and NASH [67]. This improvement in liver function could be due to a decrease in SIBO and/or dysbiosis and consequently a minor metabolic endotoxemia in the host because the restoration of normal gut microbiota might reduce intestinal permeability.

#### 2.4.2. Synbiotics

The translocation of products derived from bacteria such as lipopolysaccharide (LPS), ethanol and SCFA leads to their arrival to the liver from the intestinal lumen. Additionally, SCFAs stimulated the synthesis and storage of hepatic triacylglycerols. This process can saturate the detoxification mechanisms of the liver, resulting in an accumulation of intrahepatic triacylglycerol (IHTG) content, thus increasing the fatty liver severity. Recently, a randomized study on the use of a synbiotic that contains five probiotics (*L. plantarum*, *L. delbrueckii* spp. *bulgaricus*, *L. acidophilus*, *L. rhamnosus*, *B. bifidum* and inulin) over 6 months in adults with NASH produced a significant decrease in IHTG [67].

On the other hand, it is well known that LPS induces pro-inflammatory cytokines, such as tumor necrosis factor (TNF)-α, which play a critical role in insulin resistance and hepatic inflammatory cell recruitment in NAFLD. In fact, the evaluation of supplementation with a synbiotic, which is a mixture of *L. casei*, *L. rhamnosus*, *S. thermophilus*, *B. breve*, *L. acidophilus*, *B. longum*, *L. bulgaricus* and fructo-oligosaccharides, in a study with 52 adults over 28 weeks, demonstrated that synbiotic supplementation inhibited NF-κB and reduced TNF-α production [65]. These authors indicated an important limitation in their study, as they did not evaluate the gut microbiota to confirm the mechanism of action suggested. Furthermore, these results are still controversial because similar studies did not detect significant modifications in the values of TNF-α following treatment with several probiotics [60,61] and different synbiotics [63,67]. This could be explained by the large differences in several variables observed in the different studies, including the intervention period, the probiotic doses, and the bacterial strains used as well as the study subjects.

In summary (Table 4), some probiotics produced positive effects, mainly by improving liver function and decreasing SIBO. In the case of some synbiotics, the results showed a reduction in liver fat and TNF-α production to prevent NAFLD and its progression.

## 3. Methodology

A comprehensive search of the relevant literature was performed in electronic databases, including MEDLINE (PubMed), EMBASE and Cochrane Library. MEDLINE through PubMed was searched for human clinical trial articles that were published between 2000 and 2016 in English using the MeSH terms “probiotics” and “synbiotics” combined with “obesity”, “insulin resistance”, “diabetes mellitus, type 2”, “non-alcoholic fatty liver disease” and “metabolic syndrome X”. Here, we evaluate results obtained using the following equation search: (“obesity”[All Fields] OR “insulin resistance”[All Fields] OR “metabolic syndrome X”[All Fields] OR “non-alcoholic fatty liver disease”[All Fields] OR “diabetes mellitus, Type 2”[All Fields]) AND (“probiotics”[All Fields] OR “synbiotics”[All Fields]) AND Clinical Trial[ptyp], which yielded 66 articles. Only were included the human clinical studies regarding to obesity, IRS, T2D and NAFLD participants. A total of 36 articles were selected and revised. Additionally, we searched the reference lists of the included studies for potential relevant literature.

## 4. Conclusions

The present review focuses on the clinical effects that support the use of probiotics as a coadjuvant strategy for the prevention and treatment of obesity, IRS, T2D and NAFLD. The current scientific evidence regarding to overweight and obese patients that received some probiotics and synbiotics shows a significant reduction in the abdominal adiposity and BMI; and also, probiotic supplementation produced an improvement in the metabolism of carbohydrates, as well as a reduction in the metabolic stress in patients with T2D and IRS. Moreover, an improved serum lipid profile was observed in patients with T2D after the consumption of synbiotics. The effects of probiotics in patients with NAFLD were primarily an improvement in the liver function and metabolic parameters. Some clinical results reported beneficial effects using both probiotic and synbiotic supplementation. However, in this review, we discussed some studies that did not show any significant effect of probiotic administration on these chronic diseases. These contradictory effects in the reported studies might be related to inappropriate design such as diversity, the use of several strains, and the small number of individuals receiving some interventions.

Undeniably, further studies to evaluate the best dose-response effect of probiotics and synbiotics are needed, including following up with patients after the probiotic intervention to evaluate the persistence of their potential beneficial effects in obesity, IRS, T2D, and NAFLD.

## Figures and Tables

**Table 1:** Summary of randomized clinical intervention trials of probiotics and synbiotics in obesity.

Reference	Subjects	Strain/Dose	Time	Main Outcome
**Probiotics**				
Larsen *et al.*, 2013 [31]	50 obese adolescents	*L. salivarius* Ls-33	12 wk	Increase in the ratios of Bacteroides*, Prevotellae* and *Porphyromonas.*
Gobel *et al.*, 2012 [32]	50 adolescents with obesity	*L. salivarius* Ls-33, 10^10^ CFU	12 wk	No effect.
Kadooka *et al.*, 2010 [33]	87 subjects with high BMI	*L. gasseri* SBT2055, 5 × 10^10^ CFU	12 wk	Reduction in BMI, abdominal VFA. Increase in adiponectin levels.
Kadooka *et al.*, 2013 [34]	210 adults with large VFA	*L. gasseri* SBT2055, 10^8^ CFU	12 wk	Reduction in BMI, waist, abdominal VFA and hip circumference.
Sharafedtinov *et al.*, 2013 [35]	40 adults with obesity	*L. plantarum* 1.5 × 10^11^ CFU/g	3 wk	Reduction in BMI and arterial BP values.
Zarrati *et al.*, 2013a, 2013b, 2014 [36,37,38]	75 subjects with high BMI	*L. acidophilus* La5, *B. lactis* Bb12, and *L. casei* DN001, 10^8^ CFU/g	8 wk	Changes in gene expression in PBMCs as well as BMI, fat percentage and leptin levels.
Agerholm-Larsen *et al.*, 2000 [39]	70 overweight and obese subjects	*E. faecium* and two strains of *S. thermophilus*	8 wk	Reduction in body weight, systolic BP, LDL-C, and increase on fibrinogen levels.
Rajkumar *et al.*, 2013 [40]	60 overweight subjects	Bifidobacteria, lactobacilli, and *S. thermophilus*	6 wk	Improvement in lipid profile, insulin sensitivity, and decrease in CRP.
Brahe *et al.*, 2015 [41]	58 obese PM women	*L. paracasei* N19, 9.4× 10^10^ CFU	6 wk	No effect.
Ivey *et al.*, 2014, 2015 [42,43]	156 overweight adults	*L. acidophilus* La5 and *B. animalis* subsp. *lactis* Bb12	6 wk	Reduction in fasting glucose concentration and increase in HOMA-IR.
**Synbiotics**				
Sánchez *et al.*, 2014 [12]	153 obese men and women	*L. rhamnosus* CGMCC1.3724, 6 × 10^8^ CFU, and inulin	36 wk	Weight loss and reduction in leptin. Increase in *Lachnospiraceae*.
Safavi *et al.*, 2013 [44]	70 children and adolescents with high BMI	*L. casei*, *L. rhamnosus*, *S. thermophilus*, *B. breve*, *L. acidophilus*, *B. longum*, *L. bulgaricus*, and FOS	8 wk	Decrease in BMI *z*-score and waist circumference.
Ipar *et al.*, 2015 [45]	77 obese children	*L. acidophilus*, *L. rhamnosus*, *B. bifidum*, *B. longum*, *E. faecium*, and FOS	4 wk	Changes in anthropometric measurements. Decrease in TC, LDL-C and total oxidative stress serum levels.

Abbreviations: BMI, body mass index; BP, blood pressure; CFU, colony-forming-unit; CRP, C-reactive protein; FOS, fructo-oligossacharides; HOMA-IR, homeostasis model assessment of insulin resistance; LDL-C, low-density lipoprotein cholesterol; PBMC, peripheral blood mononuclear cell; PM, postmenopausal; TC, total cholesterol; VFA, visceral fat area; wk, week.

**Table 2:** Summary of randomized clinical intervention trials of probiotics and synbiotics in insulin resistance syndrome.

Reference	Subjects	Strain/Dose	Time	Main Outcome
**Probiotics**				
Leber *et al.*, 2013 [46]	28 patients with IRS	*L. casei* Shirota, 3 × 6.5 × 10^9^ CFU	12 wk	No effects.
Tripolt *et al.*, 2012 [47]	30 patients with IRS	*L. casei* Shirota	12 wk	Significant reduction in thesVCAM-1 level.
Barreto *et al.*, 2014 [48]	24 PM women with IRS	*L. plantarum*	12 wk	Glucose and homocysteine levels were significantly reduced.
**Synbiotics**				
Eslamparast *et al.*, 2014 [49]	38 subjects with IRS	*L. casei*, *L. rhamnosus*, *S. thermophilus*, *B. breve*, *L. acidophilus*, *B. longum*, *L. bulgaricus*, and FOS	28 wk	The levels of fasting blood sugar and insulin resistance improved significantly.

Abbreviations: CFU, colony-forming-unit; FOS, fructo-oligossacharides; IRS, insulin resistance syndrome; sVCAM-1, soluble vascular cell adhesion molecule-1; wk, week.

**Table 3:** Summary of randomized clinical intervention trials of probiotics and synbiotics in type 2 diabetes.

Reference	Subjects	Strain/Dose	Time	Main Outcome
**Probiotics**				
Hariri *et al.*, 2015 [50]	40 patients with T2D	*L. plantarum* A7	8 wk	Decreased methylation process, SOD and 8-OHDG.
Tonucci *et al.*, 2015 [51]	45 patients with T2D	*L. acidophilus* La-5 and *B. animalis* subsp. *lactis* BB-12	6 wk	Significant difference between groups concerning mean changes of HbA1c, TC and LDL-C.
Mohamadshahi *et al.*, 2014 [52]	44 patients with T2D	*L. acidophilus* La-5 and *B. animalis* subsp. *lactis* BB-12	8 wk	Increased HDL-C levels and decreased LDL-C/HDL-C ratio.
Ejtahed *et al.*, 2012 [53]	64 patients with T2D	*L. acidophilus* La5 and *B. lactis* Bb12	6 wk	Reduced fasting blood glucose and antioxidant status.
Ejtahed *et al.*, 2011 [54]	60 patients with T2D	*L. acidophilus* La5 and *B. lactis* Bb12	6 wk	TC and LDL-C improvement.
Andreasen *et al.*, 2010 [55]	45 males with T2D	*L. acidophilus* NCFM	4 wk	No effect.
**Synbiotics**				
Asemi *et al.*, 2013 [56]	54 patients with T2D (35–70 years)	*L. acidophilus*, *L. casei*, *L. rhamnosus*, *L. bulgaricus*, *B. breve*, *B. longum*, *S. thermophilus*, 10^9^ CFU, and 100 mg FOS	8 wk	Increased HOMA-IR and TGL plasma level; reduced CRP in serum.
Tajadadi-Ebrahimi *et al.*, 2014 [57]	81 patients with T2D	*L. sporogenes,* 1×10^8^ CFU and 0.07 g inulin per 1 g	8 wk	Significant reduction in serum insulin levels, HOMA-IR, and homeostatic model assessment-β-cell function.
Shakeri *et al.*, 2014 [58]	78 patients with T2D	*L. sporogenes,* 1×10^8^ CFU and 0.07 g inulin per 1 g	8 wk	Decrease in serum lipid profile (TAG, TC/HDL-C) and a significant increase in serum HDL-C levels.
Moroti *et al.*, 2012 [59]	20 patients with T2D	*L. acidophilus* 10^8^ CFU/mL, *B. bifidum* 10^8^ CFU/mL and 2 g oligofructose	2 wk	Increased HDL-C and reduced fasting glycemia.

Abbreviations: 8-OHDG; 8-hydroxy-2′-deoxyguanosine; CFU, colony-forming-unit; CRP, C-reactive protein; FOS, fructo-oligossacharides; HDL-C, high-density lipoprotein cholesterol; HOMA-IR, homeostasis model assessment of insulin resistance; LDL-C, low-density lipoprotein cholesterol; SOD, superoxide dismutase, T2D, type 2 diabetes; TAG, triacylglycerols; TC, total cholesterol; TGL, total glutathione levels; wk, week.

**Table 4:** Summary of randomized clinical intervention trials of probiotics and synbiotics in non-alcoholic fatty liver disease.

Reference	Subjects	Strain/Dose	Time	Main Outcome
**Probiotics**				
Vajro P *et al.*, 2011 [61]	20 obese children with NAFLD	*L. rhamnosus* GG, 1.2 × 10^9^ CFU/day	8 wk	Decreased ALT and PG-PS IgAg antibodies.
Aller R *et al.*, 2011 [60]	28 adult individuals with NAFLD	*L. bulgaris* and *S. thermophilus*, 5.0 × 10^11^ CFU/day	12 wk	Decreased ALT and γ-GTP levels.
Nabavi *et al.*, 2014 [62]	72 patients with NAFLD	*L. acidophilus* La5 and *B. breve* subsp. *lactis* Bb12	8 wk	Reduced serum levels of ALT, ASP, TC, and LDL-C.
Alisi A *et al.*, 2014 [63]	44 obese children with NAFLD	Bifidobacteria, lactobacilli, and *S. thermophilus*	16 wk	Improved fatty liver severity, decreased BMI and increased GLP1/aGLP1.
**Synbiotics**				
Wong VW *et al.*, 2013 [67]	20 individuals with NASH	*L. plantarum*, *L. delbrueckii* spp. *bulgaricus*, *L. acidophilus*, *L. rhamnosus*, *B. bifidum* and inulin	26 wk	Decreased IHTG content.
Eslamparast T *et al.*, 2014 [65]	52 adult individuals with NAFLD	*L. casei*, *L. rhamnosus*, *S. thermophilus*, *B. breve*, *L. acidophilus*, *B. longum*, *L. bulgaricus*, and FOS	30 wk	Inhibition of NF-κB and reduction of TNF-α.

Abbreviations: ALT, alanine amino transferase; ASP, aspartate amino transferase; CFU, colony-forming-unit; FOS, fructo-oligossacharides; γ-GTP, γ-glutamyltranspeptidase; GLP1, glucagon-like peptide 1; IHTG, intrahepatic triacylglycerol, LDL-C, low-density lipoprotein cholesterol; NAFLD, non-alcoholic fatty liver disease; NF-κB, nuclear factor κB; TC, total cholesterol; TNF-α, tumor necrosis factor α; wk, week.
